# The experiences of mine workers with cancer

**DOI:** 10.4102/hsag.v23i0.1176

**Published:** 2018-11-22

**Authors:** Portia Ramashia, Heather A. Lawrence, Fatima Bhyat

**Affiliations:** 1 Department of Medical Imaging and Radiation Sciences, University of Johannesburg, South Africa

## Abstract

**Background:**

Cancer is a disease that instils a fear of death in the minds of most people. For South African mine workers, the fear of death is compounded by a fear of being unable to fulfil work obligations in an industry where employment is central to the miners’ identity.

**Aim:**

The purpose of this research was to explore and describe the experiences of mine workers experiencing a cancer diagnosis requiring radiation therapy.

**Setting:**

Mining towns in the Limpopo province, Thabazimbi and Lephalale.

**Methods:**

A qualitative, descriptive and exploratory study design was utilised. The purposeful sample consisted of 11 mine workers receiving treatment at a radiotherapy centre in the North West province. Data were collected using an open-ended questionnaire and individual in-depth telephonic interviews. Data were analysed using open coding to identify themes.

**Results:**

The themes identified were the emotional experience resulting from the diagnosis, changing family dynamics and information needs from radiotherapy professionals. The psychosocial support required by this group of patients is unique and radiation therapists need to provide wholistic support that is tailored to address the contextual needs of this group of patients.

**Conclusions:**

Mine workers often live far away from their family and are forced to face the cancer journey alone without family support. Oncology professionals, therefore, need to create supportive structures, including emotional and financial counselling, to ensure compliance with treatment protocols, thus facilitating a positive treatment outcome.

## Introduction

Cancer is a disease dreaded by most and usually instils a fear of death in those for whom a diagnosis is made. Worldwide, cancers as a group account for 8% of all deaths annually. In South Africa, cancers as a group ranked second to cardiovascular disease amongst the elderly and account for over 15% of all deaths in the country (Bradshaw et al. 2007:643; Mqoqi et al. 2004:11; Rosen, Rodriguez-Wallberg & Rosenzweig 2009:268). It is then not surprising that a cancer diagnosis is devastating to both the patient and their family. The distress is caused by the cancer diagnosis, the patient’s sense of helplessness, uncertainty for the future and the negative physical and psychological changes associated with the treatment thereof (Bultz et al. 2011:464; Costa-Renquena & Gil 2009:187). Modern cancer treatment protocols are multi-modal in nature and result in significant acute and chronic side effects that result in physical and psychological changes in the patients. Radiotherapy is often part of the multi-modal treatment and can result in local and systemic side effects that impact the patient’s quality of life (Mason et al. 2016:221).

Mine workers in the North West province are often the main breadwinner in the family and their employment in the mining industry provides the family with an income, a medical aid and often a home. Traditionally, mine workers leave behind their families to work and live on sites in a mining camps and mining thus becomes a large part of their identity (Demissie 1998:445). Miners have had a turbulent history in South Africa and have been exploited since the early 1900s. The infamous strike of mine workers in 1946 arguably changed the political thinking of the South African liberation movement at the time and has been said to have had repercussions such as intense persecution of workers’ organisations which continue until today (South African History Online). South African miners can, therefore, be considered to be a unique group who work under difficult and often dangerous conditions with a long history of workers’ organisations that play a significant role in South African politics (Cairncross, Kisting & London 2016:513).

The North West province is serviced by only one oncology centre offering radiotherapy services, which is based in Rustenburg. Rustenburg is home to two of the largest platinum mines in the world and to the biggest producer of chrome in the world. Conditions of employment include the provision of a subsidised private medical scheme but do not always include paid sick leave that is sufficient to cover an oncology treatment protocol.

Radiotherapy requires daily attendance over a number of weeks, which may interfere with the mine workers’ ability to fulfil their work responsibilities. In the mining context, not fulfilling work responsibilities is synonymous with strike action and creates turmoil in lives of the mine workers. In the mining sector, labour unions address the needs of the group and individuals routinely abide by group action rather than by addressing individual needs. The uncertainty that is associated with a cancer diagnosis and the fear of losing mining employment because of absence can, thus, impact the patient’s compliance with oncology treatment protocols.

Radiotherapy is delivered by radiation therapists whose role includes delivering the radiation dose accurately and providing patient care, which includes effective communication, counselling and giving emotional support to patients. Traditionally patient care in radiotherapy concentrates on providing emotional support explaining procedures and responding empathetically to patient’s concerns. The mine worker as a radiotherapy patient presents a unique challenge for radiation therapists as this group of patients experience their employment in the mining sector as central to their identity and fulfilling their work responsibilities appears to be of greater concern than complying with radiotherapy treatment. There is a lack of literature that addresses this unique context from an insider perspective which would provide some insight for radiation therapists when counselling this group of patients.

### Problem statement

Radiotherapy treatment protocols require strict adherence to daily treatment attendance over several weeks and can result in acute side effects that impact on quality of life. Radiotherapy patients are encouraged to work together with radiation therapists and oncologists to manage side effects of their treatment so that the dose of radiation required can be delivered as intended. Unintentional breaks in treatment are associated with reduced overall survival rates. An understanding of the patient’s circumstances and context is required to provide empathetic and supportive practical advice to patients. There is a lack of literature that addresses the unique context of the mine worker as a radiotherapy patient. This leaves a gap in the radiation therapist’s ability to facilitate effective patient support in this group of patients, which can only be addressed by research that seeks to explore the experiences of this group.

### Research objectives

The objective of this research was to explore and describe the experiences of mine workers experiencing a cancer diagnosis requiring radiation therapy.

### Significance of the study

The significance of the study was to provide novel data on the experiences of mine workers who have a cancer diagnosis and require radiotherapy. As this group of patients presents a unique context, a need arises to ensure that this group is provided with effective patient care within the radiotherapy context.

## Research method and design

### Design

A qualitative, exploratory, descriptive design was utilised. Qualitative research designs allow the researcher to explore life experiences in order to gain insight into and understanding of phenomena (Brink, Van der Walt & Van Rensburg 2006:113; Burns & Grove 2005:52). The research design allowed a description of what it meant to experience a cancer diagnosis that required radiotherapy from the perspective of a patient whose identity lies firmly entrenched with their employment in the mining sector and for whom absence from work is synonymous with strike and protest action. Furthermore, the design allowed for information to be gathered in order for participants to identify and report their needs which can affect change in clinical practice (Holloway & Wheeler 2010:158).

### Population and sampling

The population for this research constituted mine workers receiving radiation therapy in the North West province. The participants were miners employed in the surrounding platinum mines and from neighbouring mining towns such as Thabazimbi and Lephalale in the Limpopo province. All of the participants were treated daily on an out-patient basis. The mines provided daily transport to and from the oncology centre. Purposeful sampling was used to select participants. This method ensured that information-rich participants were selected to provide information specific to the central theme of the research (Holloway & Wheeler 2010:138). Criteria for the purposeful sampling of the participants were that they were employed in the mining sector, had received radiotherapy and were willing to discuss their experiences in an interview. The researcher spent time in the oncology centre in order to develop a rapport with patients, which allowed her to identify information-rich participants. The sample size was determined by data saturation.

The total number of participants was 11, which included three females and eight males. The age ranged between 32 and 73 years. The diagnosis included cancer of the breast (3), prostate (2), lung (2), oesophagus (3) and larynx (1).

None of the participants had a tertiary education, but all were able to read and write to a satisfactory level.

### Data collection method

Data were collected during a difficult time in the mining sector which was experiencing an extended and volatile strike. The safest method to collect data was, therefore, to allow the participants to record their experiences by providing them with four open-ended questions upon which they had to reflect. This was then followed up with individual telephonic interviews as the participants did not feel safe enough to leave the mine to attend an interview. The open-ended questions were given to participants at the end of their radiotherapy treatment and the resultant responses were used to develop probing interview questions. Interviews were arranged at times that were convenient for the participants. Participants were asked to share their stories of how they experienced their diagnosis and radiotherapy treatments. Participants were also probed for information about how their experiences impacted on their role within the family unit and how the radiation therapist could assist in providing support during the treatment process. The participants were allowed to share their experiences in a language known to both the researcher and the participant. The interviews were recorded and were transcribed by the researcher. Interviews not conducted in English were translated into English by the researcher and were shared with the participants for accuracy of the translation.

During the interviews, a process of bracketing of preconceived ideas was followed by the researcher to avoid bias in the research. This process was carried out by describing her experiences of working with miners as patients to the research supervisors. Descriptive and reflective field notes were compiled during and after each interview and were used to describe possible themes and categories that emerged.

## Data analysis and results

The data analysis process included coding the raw data and finding themes, which generate recognizable patterns. The researcher listened to the interview data and read the transcripts and field notes. The data were organised and categorised into segments for the generation of meaningful codes. Common or similar codes were grouped together into categories and then reduced into broad themes based on the participants’ stories and the personal experiences of the researcher. The themes were interpreted by contextualising the descriptions with that of current literature, which addressed topics related to the theme identified (Holloway & Wheeler 2010:165–167). The themes and categories are presented in Table 1.

**TABLE 1:** Themes and categories.

Themes	Categories
1.The emotional experience that comes with a cancer diagnosis	Emotions resulting from being informed about the diagnosis Fear of death
2.Changes that mine workers and their families have to deal with after a cancer diagnosis	Family reaction to the diagnosis The effect of the diagnosis on family roles and relationships The effect of the cancer diagnosis on the mineworkers’ physical abilities
3.The information mine workers need from radiotherapy professionals	The mineworkers’ expectation of radiotherapy professionals

### Trustworthiness

The model of Lincoln and Guba (1985:290) was used to ensure the trustworthiness of this study. This model identifies the four criteria of truth-value, applicability, consistency and neutrality (De Vos 2011:443–444). Truth value was ensured by prolonged engagement with the participants before the data collection process began and by conducting peer reviews to collaborate themes. Applicability was achieved by providing sufficient background information about the study for transferability of the findings. Dependability is achieved by triangulation of data collection methods, a detailed description of the research procedure and peer review to collaborate research themes. Neutrality is ensured by providing a dependable audit trail in the form of storing raw data safely and securely and a detailed description of research procedure and process followed. Triangulation using three data collection methods (open-ended questions, individual interview and field notes) was used to ascertain reliability and validity of the study. Peer reviews were also conducted with the research supervisors to collaborate the emergent themes from the raw data. Participants were also asked to review, validate and verify the researcher’s interpretation of their experiences (Murphy & Yielder 2009:4). This was done after transcription and analysis of the unstructured interviews.

### Ethical considerations

Ethical permission to undertake the research was granted by the academic ethics research committee of the Faculty of Health Sciences at the University of Johannesburg (ethical clearance no. AEC24-01-2013). The ethical principles of respect for autonomy, non-maleficence, beneficence and justice were adhered to throughout the research process (Dhai & Mcquoid-Mason 2011:43–44). The participants’ autonomy was respected by inviting participation and obtaining consent before data were collected from each participant. Autonomy was protected by not identifying participants when reporting on the results. The participants were free to withdraw from the research process. Participants benefited from the research process by being given an opportunity to share their experiences in a positive supportive environment.

## Discussion of results

Data analysis resulted in the identification of three research themes: the emotional experiences that come with a cancer diagnosis, changes experienced by mine workers and their families, and information needs from radiotherapy professionals.

### The emotional experiences that come with a cancer diagnosis

Emotional reactions experienced after a cancer diagnosis, such as distress and fear, have been widely reported in the literature (Bultz et al. 2011:464; Costa-Renquena & Gil 2009:187). Distress could be caused by the anticipation of pain, death, social isolation, debilitating treatment regimens and diminished quality of life that are synonymous with a cancer diagnosis (Boyes et al. 2011:187). The verbatim quotes below reflect the experiences of the participants in the study.

‘The experience has been an emotional rollercoaster. As men of God I found myself asking him the one question I always preach that people must not ask God. In front of the doctor I sat there and look up the ceiling and I asked why me God.’ (Participant 1, male, miner supervisor)

My experience with the diagnosis was very emotionally challenging. I cannot even express the emotions I felt. I remember my eyes filled with tears. (Participant 4, male, mine worker)

As soon as he said cancer, the first thing I thought was: I can’t die now, and then I didn’t think or hear anymore of what was said. He might have said that my type of cancer was possible to cure, but I simply didn’t catch it. I was worried about my family and how they were going to carry on when I am dead. (Participant 3, female, human resource employee)

Bultz et al. (2011:464), Hinz et al. (2009:369) and Hill et al. (2011:1432) have shown that the news of a cancer diagnosis is experienced negatively by the majority of newly diagnosed cancer patients. Receiving bad news usually creates a crisis for the patient, often manifested by intense anxiety, uncertainty, confusion, helplessness and fear of losing control over one’s life (Buckman 1992:114). Furthermore, the expression of a wide range of emotions, including sadness, anger, despair or gallows humour (Baile & Beale 2001:2576), is regularly reported. The emotions expressed by the participants in the study were in line with typical emotions reported in the literature and did not seem to be compounded by their identity formed by working and living in the mining industry.

### Changes experienced by mine workers and their families

Cancer is a family experience. Family members often provide meaningful support to the patient and act as the ‘natural’ caregivers, complementing the skills of the professional healthcare team (Papastavrou, Charalambous & Tsangari 2009:135). The participants in this study reported feeling scared, uncertain and angry about the unwanted changes cancer would bring into their lives. Unique to the mining setting is the phenomenon of the cancer patient whose family does not live with them at the mine. The family is still impacted by the diagnosis but does not necessarily understand why the changes in the family circumstances occur. In this context, the family is unable to provide emotional and physical support to the cancer patient. Typical responses expressed by participants are illustrated by the following verbatim quotations:

‘Because of the cancer and this long treatment I am not getting my full salary…jaa, I sometimes think of stopping the treatment because as it is I have not been able to go visit my family in the village. My family don’t believe that I don’t have money; my wife thinks I am using the money on my girlfriends. I don’t know which girlfriend will want a skeleton [*lost weight*] like me.’ (Participant 4, male, mine worker)

‘My family just know I am sick, they don’t know what disease I have. Myself I don’t even understand, I just told them the Doctor says I have cancer and I am going for treatment everyday; I had to tell them because of the salary that I am not getting in full. My wife must just take care of the kids while I get the treatment; hopefully I will survive this and get back to normal and play my role in the family as a man.’ (Participant 5, male, mine worker)

The verbatim quotes above are unique to this context and are inconsistent with the literature, which normally details the experiences of patients who are living with their families. However, participants who were living in the mines with their families reported changes in family dynamics that are more consistent with the literature. Examples of such experiences are described below:

‘I have a sister and sister-in-law that have been so supportive of me. They made a statement when I was diagnosed; they said, “Well, you do not have a cancer, we have a cancer, and we will work through it”. And now, we are on treatment. ‘(Participant 2, female, lamp room controller)

‘My family was more devastated than me, I actually thought they thought I was going to die the very next day and we got closer as a result of the diagnosis, and family started spending more time around me and my two girls. (Participant 3, female, human resource employee)

‘My wife was always very dependent on me. I was very hurt the other day when I overheard her asking her brother about filling up the car because she had no idea. From where I was I could hear she was crying saying “my husband has always done that” it really hurt me to hear her saying that.’ (Participant 1, male, miner supervisor)

Cancer and cancer treatments impact not only the patients but also their family and friends (Stenberg et al. 2010). Families face a set of psychosocial challenges imposed by their relative’s cancer, including coping with uncertainty, dealing with illness-related losses and adapting to the changes engendered by cancer and its treatment (Raveis 2007). When a family member becomes ill, the ramifications of the illness are experienced throughout the family system. The diagnosis of a life-threatening illness for a family member creates fear of losing the loved one and concern about the suffering he or she will endure. The family experienced being deprived of their everyday normal family life as the family life was experienced as disrupted (Raveis, Pretter & Carrero 2010:132).

### Information needs from radiotherapy professionals

Mine workers as cancer patients are a unique patient group. They typically have low levels of formal education and come from lower income groups and poor socio-economic background. English is often not their home language, yet it is the language that oncology professionals typically use when communicating with them. Culturally, mine workers are often sceptical of Western medicine and have a wider acceptance of African traditional medicine when faced with illness. Their information needs are, therefore, different from those patients typically reported in oncology literature:

‘I would have appreciated explanation on how my body was going to change with the treatment. I was also in the dark about what I can eat, I definitely think somewhere in those early stages if someone suggested seeing someone or seeing a counsellor it would have made a big difference for me and my family.’ (Participant 3, female, human resource employee)

‘When I arrived in the oncology department I was lost. I did not even know what cancer was I expected an explanation from the professionals. I think it would be nice to have someone, I don’t know a social worker whatever but who knew about individual cancers.’ (Participant 5, male, mine worker)

‘It would have been nice if somebody had said: Is everything going alright for you, have you got support at home.’ (Participant 2, female, lamp room controller)

‘They must arrange with the Mine to pay us full pay even if we are not working.’ (Participant 4, male, mine worker)

Information is vital for patients and their families to make choices and decisions in their efforts to seek a cure and, when this is not possible, to cope with the disease and all that it brings (McCaughan & McKenna 2007:2097). Unnecessary anxieties and inappropriate responses can be created by lack of information arising from the myths, misconceptions and uncertainties surrounding cancer. The above quotations seem to indicate that information that may have been given to the participants was not fully understood by the participants and was perhaps given at a level inconsistent with their knowledge of cancer and their education levels. Furthermore, a common source of anxiety in this group of cancer patients seems to arise from financial concerns because of altered employment benefits. The reality of this is not typically addressed by oncology professionals despite the consequences such as non-compliance to treatment protocols. Carlson and Bultz (2004:840) and Longo et al. (2006:1077) have described the lowered overall functioning and altered quality of life experienced by unmet financial needs, which may inhibit compliance with treatment regimens creating increased psychosocial needs and emotional distress. Oncology professionals need to ensure that information giving promotes understanding of the disease and its treatment while caring for the patient holistically, which would include counselling patients throughout their cancer journey.

## Limitations of the study

The study was conducted with participants employed in the mining sector at a time where labour unrest resulted in a volatile setting. Data collection was impacted as participants were unwilling to leave the mine setting out of fear of missing out on communication from the employers and union leaders. Therefore, telephonic interviews were conducted and non-verbal language was not possible to record.

## Recommendations

Mine workers are a unique group of patients who require special attention to be paid to concerns that arise from their environment and culture. The mine is central to their identity, and their employment in the mining sector becomes a vital part of their cancer journey. The needs of the mine worker as a cancer patient include to be given information in an appropriate format. Limited formal education levels may result in a lack of understanding of cancer and its treatment. Oncology professionals become the sole source of information for many of these patients and often provide information in a written format and at a level which is not appropriate for the knowledge base of the mine worker. This results in many patients experiencing treatment for a disease they do not understand and experiencing side effects for which they are unprepared. A recommendation for oncology professionals would be to provide regular information-giving sessions to this group of patients in a language that they understand and at a level that is appropriate.

Furthermore, the psychosocial support required by this group of patients is unique. Support groups should be developed to replace the support traditionally given by the family of the cancer patient. Financial counselling and a supportive relationship with mine employers should be encouraged to enable an environment to be created where compliance to treatment protocols is not compromised by fear of losing mine employment and financial stability.

## Conclusion

This research aimed to explore and describe the experiences of mine workers experiencing a cancer diagnosis requiring radiation therapy. The results showed that mine workers rely on healthcare professionals to get information about their diagnosis and treatment at an appropriate level, who often become the sole source of information for this group of patients. A supportive environment that includes emotional and financial counselling is important to ensure compliance with treatment protocols. Mine workers are often living away from family and face the cancer journey alone without family support. Oncology professionals need to create a supportive structure that could enable the holistic management of the patient and facilitate a positive treatment outcome.
